# Low-dimensional gap plasmons for enhanced light-graphene interactions

**DOI:** 10.1038/srep43333

**Published:** 2017-02-27

**Authors:** Yunjung Kim, Sunkyu Yu, Namkyoo Park

**Affiliations:** 1 Photonic Systems Laboratory, Department of Electrical and Computer Engineering, Seoul National University, Seoul 08826, Korea

## Abstract

Graphene plasmonics has become a highlighted research area due to the outstanding properties of deep-subwavelength plasmon excitation, long relaxation time, and electro-optical tunability. Although the giant conductivity of a graphene layer enables the low-dimensional confinement of light, the atomic scale of the layer thickness is severely mismatched with optical mode sizes, which impedes the efficient tuning of graphene plasmon modes from the degraded light-graphene overlap. Inspired by gap plasmon modes in noble metals, here we propose low-dimensional hybrid graphene gap plasmon waves for large light-graphene overlap factor. We show that gap plasmon waves exhibit improved in-plane and out-of-plane field concentrations on graphene compared to those of edge or wire-like graphene plasmons. By adjusting the chemical property of the graphene layer, efficient and linear modulation of hybrid graphene gap plasmon modes is also achieved. Our results provide potential opportunities to low-dimensional graphene plasmonic devices with strong tunability.

In the context of light-matter interactions, the concentration of electromagnetic fields on materials is a critical issue for the performance of tunable optical devices, such as photodetectors^1^, bio-sensors^2^, optical modulators^3^,^4^, and lasers^5^. Plasmonic structures^6^,^7^,^8^,^9^ have thus been intensively studied to achieve subwavelength field concentration. For the design of plasmonic devices, the proper selection of metals determines the boundary of device performances^10^ for power consumption, bandwidth, and footprints.

Due to its two-dimensional (2D) structure with extremely large conductivity from the massless Dirac point^11^,^12^, graphene has become a leading candidate for deep-subwavelength plasmonics^13^,^14^,^15^. Along with its structural advantage for the integration, the giant and tunable conductivity of the graphene layer also enables the modulation of its optical properties. A number of devices such as absorbers^16^,^17^,^18^, modulators^19^,^20^, and tunable metamaterials^21^,^22^,^23^,^24^,^25^ controlling optical flows through the designed graphene layer have been proposed and demonstrated, by manipulating the dispersion of graphene conductivity via electric gating^21^,^26^,^27^,^28^ or chemical doping^29^,^30^. However, the atomically-thin graphene layer leads to the intrinsic limit for the device performance at the same time; while the mismatch between the wavelength of light and graphene layers hinders the excitation to graphene-based devices, the significant scale mismatch between ~10 nm to ~100 nm size optical modes and ~Å-scale graphene layers severely degrades the light-graphene overlap which prohibits the efficient manipulation of light flows in terms of light-matter interactions. The achievement of small modal size^31^,^32^ and more importantly, the high overlap factor with the graphene layer, is thus an urgent issue for tunable graphene plasmonics.

Here, we focus on low-dimensional waveguide systems for the improved light-graphene overlap factor. We firstly reveal the existence of hybrid graphene gap plasmon (H-GGP) modes the field profile of which is strongly confined inside the graphene gap between metallic and dielectric graphene layers. We demonstrate that the H-GGP mode has larger field concentration on graphene layers than those of edge^33^,^34^ or wire-like graphene plasmon modes^31^. By exploiting the tunable graphene conductivity through the chemical potential modulation, highly sensitive and linear modulation of the H-GGP propagation constant is also achieved with its stable mode profile. The proposed low-dimensional waveguide systems with the improved overlap factor pave the path toward integrated plasmonic devices on graphene.

## Results

**Figure 1:**
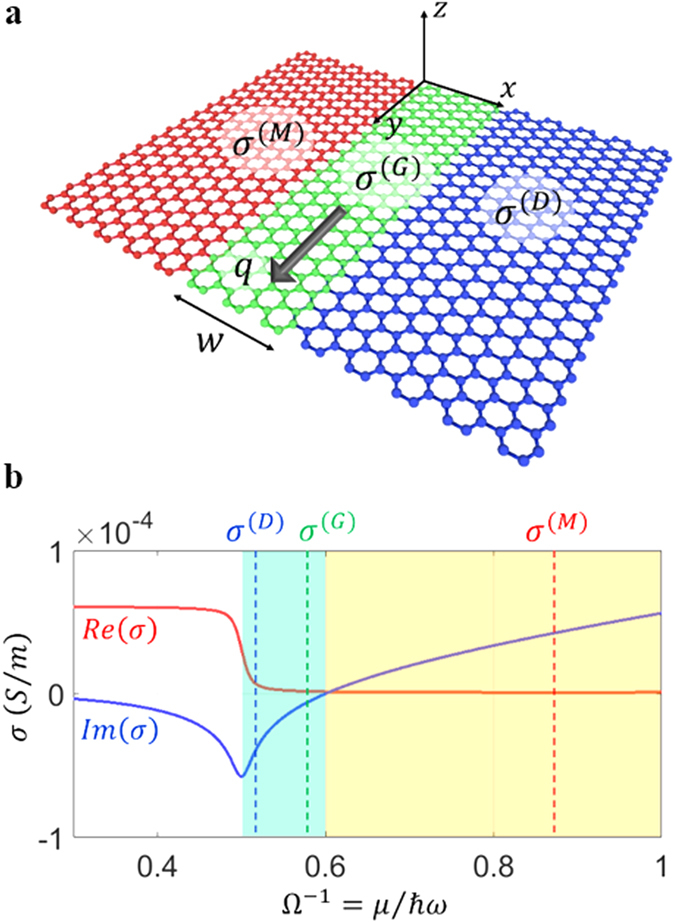
Low-dimensional (2D) graphene waveguide system for H-GGP modes, consisting of three distinct graphene domains. (**a**) A schematic of the proposed 2D metal-gap-dielectric system, composed of a gap domain G (dielectric, 0 < *x* < *w; σ*^(G)^) between two semi-infinite domains M (metallic, *x* < 0; *σ*^(M)^) and D (dielectric, *x* > *w; σ*^(D)^). The H-GGP mode is assumed to propagate along the *y*-axis with the wavevector ***q*** = q***k***. (**b**) The graphene conductivity as a function of the normalized chemical potential *Ω*^−1^, satisfying the condition of *Im*{*σ*^(D)^} < *Im*{*σ*^(G)^} < 0 < *Im*{*σ*^(M)^}. The blue-green (or yellow) region denotes the dielectric (or metallic) regime with 0.5 < *Ω*^−1^ < 0.6 (or *Ω*^−1^ > 0.6). The highly-lossy region (*Ω*^−1^ < 0.5 with *Re(σ*) > > 0) from the interband transition is excluded in our discussion. Graphene structures considered here are assumed to be suspended in air.

We consider the 2D metal-gap-dielectric waveguide system composed of three distinct graphene domains (Fig. 1a); a dielectric gap domain G with the width *w* (the sheet conductivity *σ*^(G)^ where *Im*{*σ*^(G)^} < 0) is inserted in-between semi-infinite metallic domain M (*Im*{*σ*^(M)^} > 0) and dielectric domain D (*Im*{*σ*^(D)^} < 0). The gap region G satisfies the condition of *Im*{*σ*^(D)^} < *Im*{*σ*^(G)^} < 0, as the analogy of plasmonic gap modes in noble metals^8^,^9^. Note that such a system can be achieved by applying the spatial variation of the conductivity on a single graphene layer, based on the tuning of its chemical potential^21^,^26^,^27^,^28^ (or doping level) *μ(x*). Figure 1b shows the *μ*-dependency of the graphene conductivity calculated by Kubo formula^11^,^21^,^23^, which determines the operation regime for each layer (frequency *f* = *ω*/2*π* = 20 THz, charged particle scattering rate^11^ Γ = 0.43 meV, and temperature T = 3 K). To satisfy the gap mode condition as similar to the case of noble metals^8^,^9^, the proposed system can be realized by adjusting the doping level of each graphene region corresponding to (*Ω*^(M)^)^−1^ > 0.6 and 0.5 < (*Ω*^(D)^)^−1^ < (*Ω*^(G)^)^−1^ < 0.6, where *Ω*^−1^ = *μ*/(ℏ*ω*) is the normalized chemical potential.

**Figure 2:**
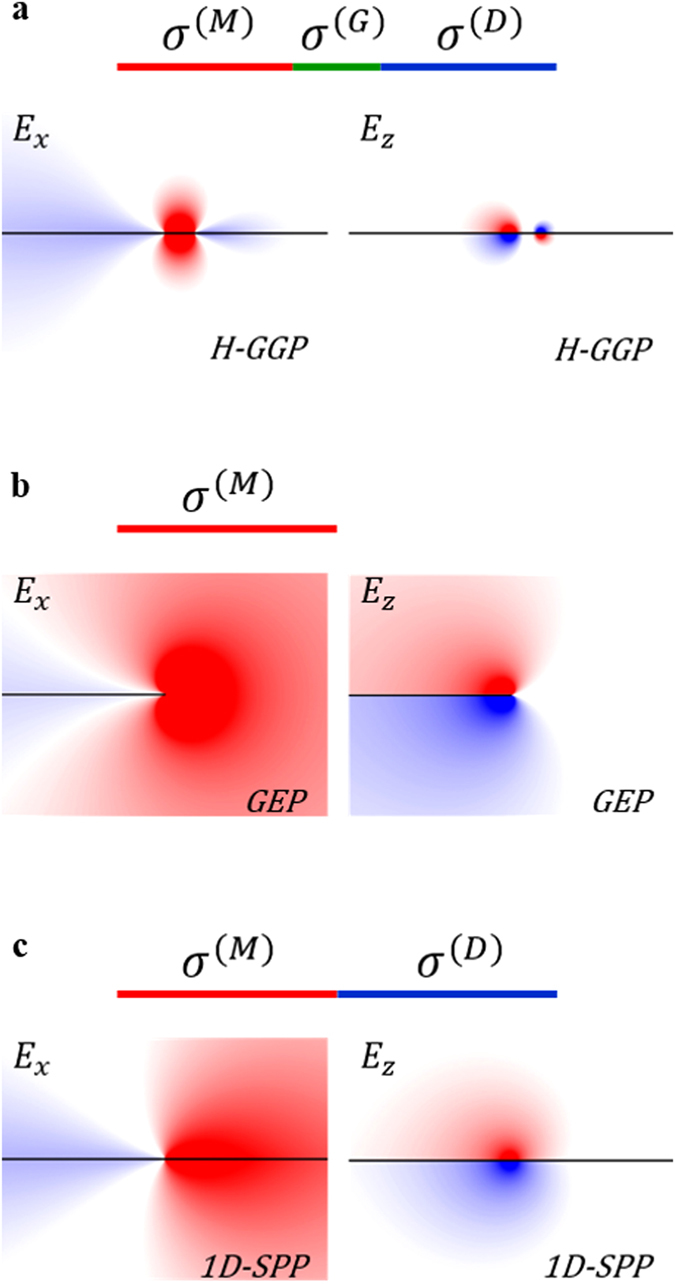
Electric field distributions of graphene plasmon modes for (**a**) H-GGP mode (*w* = 5 nm and (*Ω*^(G)^)^−1^ = 0.54), (**b**) GEP mode, and (**c**) wire-like 1D-SPP mode. (*Ω*^(M)^)^−1^ = 4 for all cases, and (*Ω*^(D)^)^−1^ = 0.5002 for (**a**,**c**). The horizontal black lines indicate graphene layers. The components of electric fields *E*_*x,z*_ are normalized for clarity.

Figure 2a shows the electric field profile of the low-dimensional H-GGP mode in the 2D metal-gap-dielectric waveguide system, calculated by the eigenmode solver of COMSOL Multiphysics (*w* = 5 nm, (*Ω*^(M)^)^−1^ = 4, (*Ω*^(G)^)^−1^ = 0.54, and (*Ω*^(D)^)^−1^ = 0.5002). In the numerical analysis, the graphene is considered as the film^21^ with the thickness^31^ of *δ* = 0.2 nm and the relative bulk permittivity of *ε*_g_(*ω*) = 1 + *jσ*_g_(*ω*)/(*ωε*_0_*δ*), where *σ*_g_ is sheet conductivity of graphene obtained from Kubo formula^11^ (Fig. 1b). To demonstrate the distinctive feature of H-GGP modes, we compare with other graphene waveguide modes: graphene edge plasmon (GEP) mode^33^,^34^ (Fig. 2b) and wire-like 1D-SPP mode^31^ (Fig. 2c) with same material parameters. While both H-GGP and 1D-SPP modes with quasi-antisymmetric potential profiles (*σ(x, z*)~ − *σ*(−*x, z*) for all *z*) have improved confinement compared to that of the GEP mode with much stronger structural asymmetry (|*σ(x*,0)| ≪ |*σ*(−*x*,0)| and |*σ(x, z*)| = |*σ*(−*x, z*)| for *z* ≠ 0), H-GGP exhibits more confined transverse (*E*_*x*_) field on the gap region than that of the 1D-SPP mode, as similar to the difference between gap plasmons and surface plasmons in noble metals^8^,^9^. This transverse concentration originates from the continuity condition of the displacement current *Im*{*σ*^(G)^}·*E*_*x*_^(G)^~*Im*{*σ*^(D)^}·*E*_*x*_^(D)^, deriving the enhancement of *E*_*x*_^(G)^ from the condition of *Im*{*σ*^(D)^} < *Im*{*σ*^(G)^} < 0. For the practical realization, we also note that H-GGP modes can be obtained at any finite temperatures when the conductivity of each domain satisfies the condition of *Im*{*σ*^(D)^} < *Im*{*σ*^(G)^} < 0 < *Im*{*σ*^(M)^}, as shown in Supplementary Note 1, demonstrating the existence of H-GGP modes in room temperature (T = 300 K), at the frequency of *f* = 100 THz.

**Figure 3:**
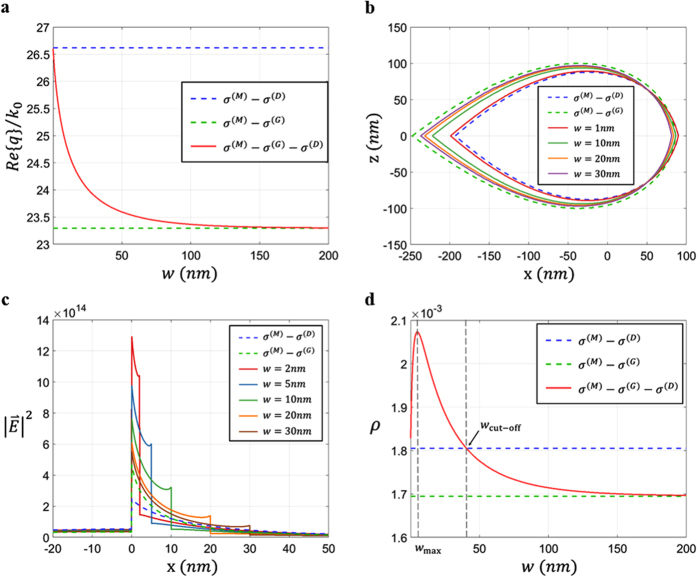
Modal properties of the H-GGP mode controlled by the structural parameter *w*. (**a**) Effective mode index *n*_eff_ = *Re*{*q*}/*k*_0_ of the H-GGP mode as a function of the gap width *w*. (**b**) Modal cross section contours corresponding to *w* = 1, 10, 20, and 30 nm, which depict the region A for ∫∫_A_|***E***|^2^·d***S***/∫∫|***E***|^2^·d***S*** = 0.8. (**c**) Electric field intensity along the center of the graphene layer (*x*-axis) for different gap widths *w*, compared to the cases of *σ*^(M)^ − *σ*^(D)^ and *σ*^(M)^ − *σ*^(G)^ 1D-SPP systems. (**d**) Graphene field concentration *ρ* as a function of the gap width *w (w*_max_ = 5 nm, *w*_cut-off_ = 40 nm). The blue dashed (green dashed) line in (**a**–**d**) denotes 1D-SPP modes. All other parameters are same as those in Fig. 2.

Figure 3 shows the characteristics of the H-GGP mode, including effective mode index, intensity profiles, and field concentration. As similar to the case of noble metal gap plasmons^35^, many aspects of the H-GGP mode represent the intermediate features between those of 1D-SPP modes for low and high dielectric graphene layers. For example, the proposed H-GGP system with zero width (*w* = 0) corresponds to the *σ*^(M)^ − *σ*^(D)^ 1D-SPP system with the effective mode index^31^ of *n*_eff_ = *Re*{*q*}/*k*_0_ ≈ 2·(3/2)^1/2^·*εε*_0_·*c*/(*Im*{*σ*^(M)^} + *Im*{*σ*^(D)^}), while the H-GGP system with infinite *w* is converged to the *σ*^(M)^ − *σ*^(G)^ 1D-SPP system. The effective mode index (Fig. 3a) and the modal size (Fig. 3b) of the H-GGP mode with different widths are thus varying between these two boundaries.

Most importantly, there exist differentiated features of the H-GGP mode when compared to 1D-SPP modes, as shown in Fig. 3c and d. Figure 3c shows the electric field intensity of the H-GGP modes along the center of the graphene layer for different gap width (2 nm to 30 nm). Although the intensity profile of large *w* case is converged to that of the *σ*^(M)^ − *σ*^(G)^ 1D-SPP system, smaller *w* cases exhibit the intensity profiles focused of the graphene gap. Such a distinct in-plane intensity distribution imposes the unique property on out-of-plane confinement, in terms of the light-graphene overlap factor *ρ* = ∫∫_graphene_|***E***|^2^·d***S***/∫∫|***E***|^2^·d***S***: the concentration of electromagnetic fields on graphene. Figure 3d presents the variation of *ρ* as a function of the gap width *w*, which demonstrates the improved light-graphene overlap for the structures with apparent field concentration on the gap (0 < *w* < 40 nm). We note that the H-GGP mode acquires much higher field concentration on the graphene layer (*ρ* = 2.07 × 10^−3^ at *w* = 5 nm), when compared to those of 1D-SPP modes (*σ*^(M)^ − *σ*^(D)^ system of *ρ* = 1.81 × 10^−3^ and *σ*^(M)^ − *σ*^(G)^ system of *ρ* = 1.70 × 10^−3^) and the GEP mode (*ρ* = 0.728 × 10^−3^).

**Figure 4:**
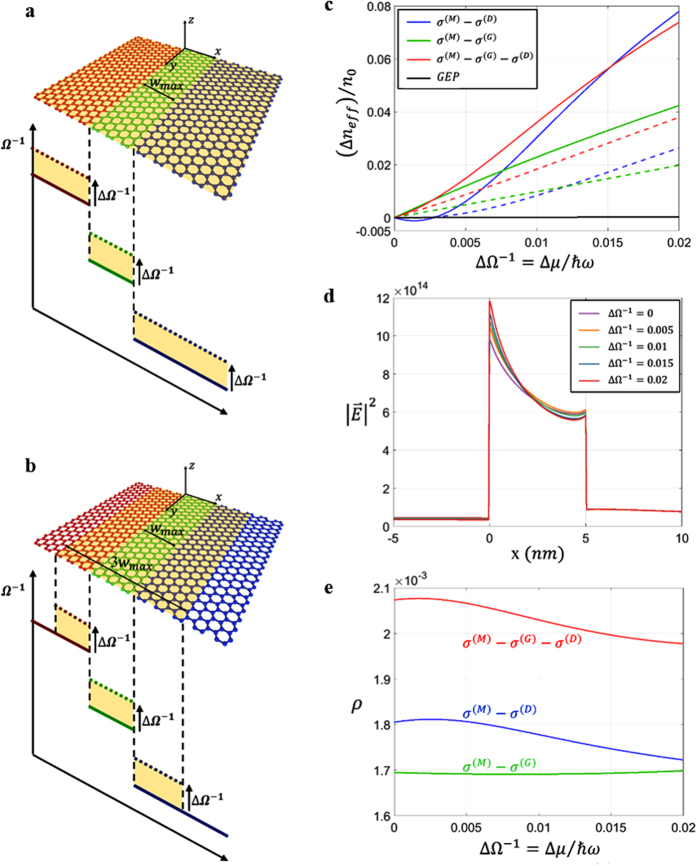
The effect of the chemical potential modulation on the characteristics of H-GGP modes. The schematics of the chemical potential modulation for cases of spatially (**a**) global and (**b**) local (3*w*_max_ modulation width) modulations on the transverse plane. Yellow regions in (**a**,**b**) indicate the modulation range. (**c**) The variation of effective mode index *n*_eff_ for H-GGP, 1D-SPP, and GEP modes for the case of *w*_max_ = 5 nm, as a function of the chemical potential modulation *ΔΩ*^−1^. Dashed lines denote the case of the spatially local modulation on the transverse plane (Fig.4b). (**d**) Electric field intensity of the H-GGP mode along the center of the graphene layer (*x*-axis), for different values of *ΔΩ*^−1^. (**e**) Graphene field concentration factor *ρ* as a function of *ΔΩ*^−1^. GEP mode exhibits much lower *ρ* in (**e**) (*ρ*~0.728 × 10^−3^, not shown). All other parameters are same as those in Fig. 2. See Supplementary Fig. 2 for the schematics of the modulation range for the cases of 1D-SPP modes (solid and dotted lines of blue and green colors).

The large overlap factor in Fig. 3 allows for the enhancement of light-graphene interactions. Figure 4 shows the modulation of H-GGP modes by controlling the chemical potential of the graphene layer as (*Ω*^(M)^)^−1^ = 4 + *ΔΩ*^−1^, (*Ω*^(G)^)^−1^ = 0.54 + *ΔΩ*^−1^, and (*Ω*^(D)^)^−1^ = 0.5002 + *ΔΩ*^−1^, with the spatially global (Fig. 4a) and local (Fig. 4b, 3*w*_max_ modulation width) modulation range. As seen, the effective mode index of the H-GGP mode can be controlled with an order of smaller modulation of *ΔΩ*^−1^ when compared to the GEP mode. The H-GGP mode also provides more efficient regime of *ΔΩ*^−1^ for controlling effective index compared to 1D-SPP modes (*ΔΩ*^−1^ ≤ 0.015). Such improved efficiency is more apparent for the case of the finite modulation region for *ΔΩ*^−1^ (dotted lines in Fig. 4c, for the 3*w*_max_ modulation width around the graphene gap), due to the improved transverse localization of the H-GGP mode (Fig. 3c). Note that the spatial profile of electric field intensity (Fig. 4d) and the overlap factor *ρ* (Fig. 4e) of the H-GGP mode is highly stable to the change of *ΔΩ*^−1^. This stability allows the adiabatic change of the propagation feature of the H-GGP mode, which is the origin of the linear variation of *n*_eff_ versus *ΔΩ*^−1^ in Fig. 4c. Because the change of chemical potentials is usually derived by the external electric field, the sensitive and linear modulation of the H-GGP mode demonstrated in Fig. 4 enables the high-speed and low-power realization of tunable graphene devices.

## Discussion

We demonstrated the existence of low-dimensional gap plasmon modes on graphene, which supports large light-graphene overlap factor. The system with spatially-varying chemical potential (or doping level) for H-GGP modes can be realized by several existing schemes such as electric field bias^21^ or substrate level control^36^. Highly efficient manipulation with the stable field profile of the H-GGP mode, improved from those of GEP^33^,^34^ or wire-like 1D-SPP modes^31^, opens the pathway toward tunable graphene plasmonics with high-speed and low-power operation. The modal profile dependency of the light-graphene overlap factor also imposes intriguing opportunity on unconventional wave profiles supported by 2D materials, based on optical transformation techniques^21^,^37^,^38^. In terms of future applications, the compensation method of the large difference between effective mode index of graphene plasmonics and that of air (*n* = 1) will be required for increasing excitation efficiency, for example, by applying adiabatic procedure or matched layers.

## Additional Information

**How to cite this article**: Kim, Y. *et al*. Low-dimensional gap plasmons for enhanced light-graphene interactions. *Sci. Rep.* **7**, 43333; doi: 10.1038/srep43333 (2017).

**Publisher's note:** Springer Nature remains neutral with regard to jurisdictional claims in published maps and institutional affiliations.

## Supplementary Material

Supplementary Information
