# Supplementary material for: Low-dimensional gap plasmons for enhanced light-graphene interactions
Source: Sci Rep. 2017 Feb 27;7:43333. doi: 10.1038/srep43333 (PMC5327386; doi:10.1038/srep43333)
Supplement: Supplementary Information [file srep43333-s1.pdf]

Low-dimensional gap plasmons for enhanced light-graphene interactions

Yunjung Kim, Sunkyu Yu, and Namkyoo Park*

Photonic Systems Laboratory, Department of Electrical and Computer Engineering, Seoul National University, Seoul 08826, Korea

**E-mail address for correspondence: nkpark@snu.ac.kr*

Supplemental Material

Supplementary Note 1. The characteristics of the H-GGP mode at room temperature

Supplementary Note 2. Schematics for spatially global and local modulations for 1D-SPP

Supplementary Figure 1. Modal properties of the H-GGP mode at T = 300K.

Supplementary Figure 2. The schematics of the chemical potential modulation for 1D-SPP modes at the $\sigma^{(M)}$-$\sigma^{(D)}$ and $\sigma^{(M)}$-$\sigma^{(G)}$ interfaces.

Supplementary Note 1. The characteristics of the H-GGP mode at room temperature

Figure 1 shows the existence of H-GGP mode at room temperature (T = 300K). The graphene conductivity calculated by Kubo formula[1-3] exhibits the dielectric characteristics ($Im\{\sigma\} < 0$) at higher frequency ($\hbar\omega \gg k_BT$) as the temperature increases[4] from T = 3K to T = 300K. Supplementary Figure 1 shows modal properties of the H-GGP mode achieved at T = 300K, $f = \omega / 2\pi$ = 100THz, $(\Omega^{(M)})^{-1} = 2$, $(\Omega^{(G)})^{-1} = 0.59$, and $(\Omega^{(D)})^{-1} = 0.5002$.

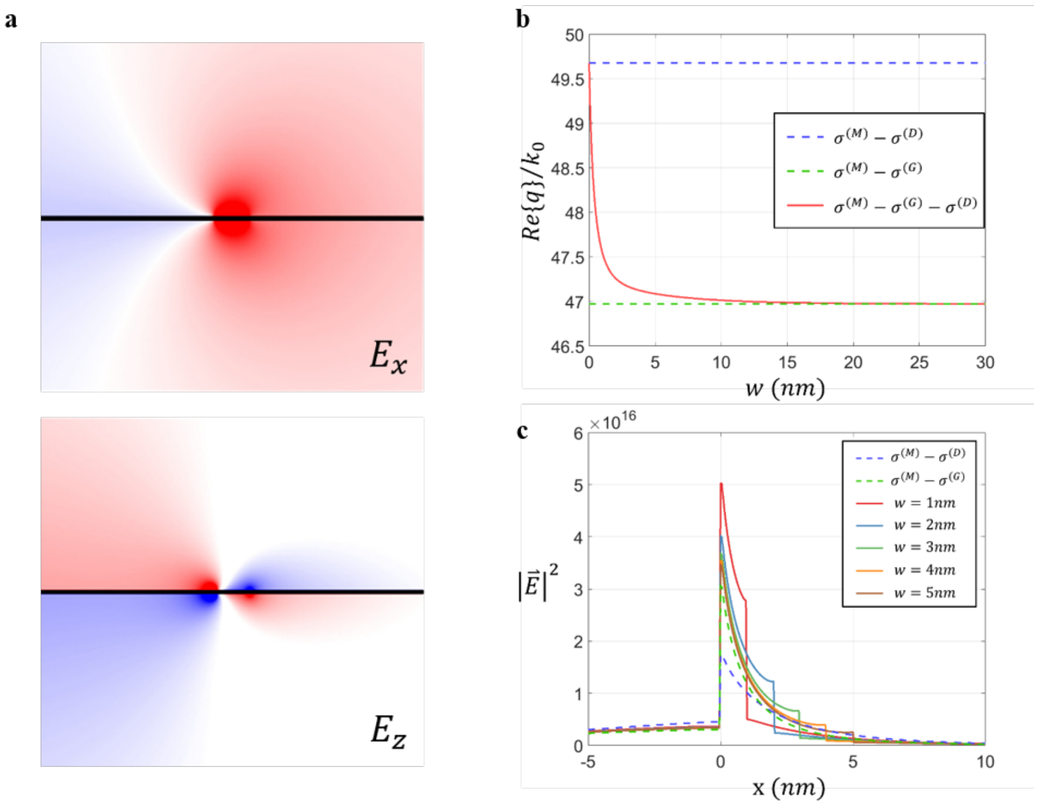

Supplementary Figure 1. Modal properties of the H-GGP mode at T = 300K. (**a**) Electric field distributions of H-GGP mode with the gap width, w = 1nm. The horizontal black lines indicate graphene layers. The components of electric fields $E_{x,z}$ are normalized for clarity. (**b**) Effective mode index $n_{\mathrm{eff}} = Re\{q\}/k_0$ of the H-GGP mode as a function of the gap width w. (**c**) Electric field intensity along the center of the graphene layer (x-axis) for different gap widths w, compared to the cases of $\sigma^{(M)}$-$\sigma^{(D)}$ and $\sigma^{(M)}$-$\sigma^{(G)}$ 1D-SPP systems.

Supplementary Note 2. Schematics for spatially global and local modulations for 1D-SPP

Supplementary Figure 2 shows the schematics of the modulation range for spatially global and local modulations of 1D-SPP modes. As shown, the case of the global modulation (Supplementary Fig. 2a) tunes the chemical potential of the overall graphene layer, whereas the local modulation (Supplementary Fig. 2b) has the finite modulation region ($3w_{\mathrm{max}}$ modulation width) around the graphene interface for the 1D-SPP mode.

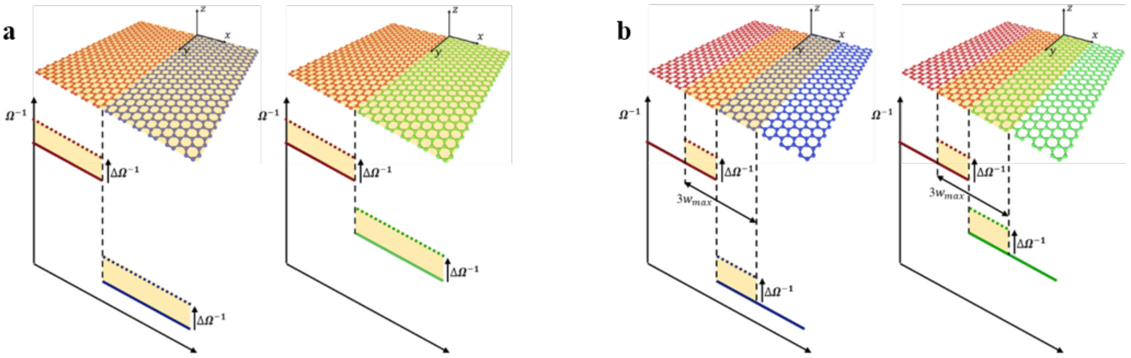

Supplementary Figure 2. The schematics of the chemical potential modulation for 1D-SPP modes at the $\sigma^{(M)}$-$\sigma^{(D)}$ and $\sigma^{(M)}$-$\sigma^{(G)}$ interfaces. (**a**) The cases of the global and (**b**) local modulations on $\sigma^{(M)}$-$\sigma^{(D)}$ (left) and $\sigma^{(M)}$-$\sigma^{(G)}$ (right) 1D-SPP systems. Yellow regions in (**a**,**b**) indicate the modulation range.

References

1. Gusynin, V. P., Sharapov, S. G. & Carbotte, J. P. Magneto-optical conductivity in graphene. *J. Phys.: Condens. Matter* **19**, 026222, doi:10.1088/0953-8984/19/2/026222 (2007).

2. Vakil, A. & Engheta, N. Transformation optics using graphene. *Science* **332**, 1291-1294, doi:10.1126/science.1202691 (2011).

3. Fallahi, A. & Perruisseau-Carrier, J. Design of tunable biperiodic graphene metasurfaces. *Phys. Rev. B* **86**, 195408, doi:10.1103/PhysRevB.86.195408 (2012).

4. Farhat, M., Rockstuhl, C. & Bagci, H. A 3D tunable and multi-frequency graphene plasmonic cloak. *Opt. Express* **21**, 12592-12603, doi:10.1364/OE.21.012592 (2013).
